# Observations on a Case of Strangulated Hernia; *Communicated in a Letter from* Dr. Chatard, *of Baltimore, to* Dr. Miller

**Published:** 1805-09-01

**Authors:** 


### Observations on a Case of Strangulated Hernia; *communicated in a Letter from* Dr. Chatard, *of Baltimore, to* Dr. Miller.

[ From the New York Medical Repository. ]

NORBERE BELAIR, surnamed the Picard, a sailor who had deserted from the French frigate Poursuivante, requested me to visit him on the 20th of last February, at five o'clock in the afternoon, on account of violent illness. I found him in bed, with high fever, occasioned by the strangulation of an inguinal hernia, which had taken place five hours before, from his exertions in loading a cart, while unprovided with his truss, which he had generally worn for the two preceding years. As this man was of a robust habit, I ordered him to be bled to twenty-five ounces, and then attempted to reduce the hernia by the *taxis*; but in this I was unable to succeed. I directed him that night to go twice into the warm bath, and to remain in it two hours at each time, and, in the intervals of the bath, to apply a bread poultice. On the 21st, at eight o'clock in the morning, he continued in the same situation, and his fever being still high, I determined on a second blood-letting, nearly as copious as the first. The baths, the poultices, the injections, were used, one after another, for twenty-four hours, with great exactness; and, in addition, I ordered a grain of opium to be given every sixth hour, to relieve the violent pain, the hiccup, and the vomiting. Every thing was unavailing; and at the beginning of the third day no change had taken place in the condition of the unhappy patient. I thought it my duty to urge the operation as the last resource; but he obstinately opposed it, and declared he would rather die than submit to it. Having much confidence in the treatment used in this case, as I had often seen it succeed under the direction of M. Desault, after persevering in it many days, I insisted on the employment of the baths, the injections, the poultices, and the opium, as before, during the six first days, but they were not used with perfect exactness; and still every day I continued to urge the operation, which was firmly and steadily rejected. At the end of the time just mentioned, the patient, who could take nothing without rejecting it from his stomach, became came extremely weak, and appeared to me to be in a desperate situation. Still, however, I continued to visit him as frequently as I could, as well as to encourage the assiduities of those to whose care he was committed, and who were beginning to grow weary of their duty, as to observe the progress and termination of a case which seemed to assume a singular character. It was not till near the close of the fifteenth day of the disease that the patient breathed his last. On the twelfth day of the disease he still had strength enough to walk from the cellar in which he lay to a kitchen situated above, and at the distance of some paces from it; where he ate eggs, meat, bread, and drank tea, with very great appetite, and retained these things on his stomach for several hours. During the whole course of the disease he only discharged by the anus the water of the injections, after those first administered had emptied the large intestines of the excremental matters contained in that portion of them which was below the strangulated part.

About the sixth day the hernial tumour seemed, by imperceptible degrees, to grow smaller, and on the twelfth I observed a fluctuation at the bottom of it, which led me to apprehend that possibly an opening might take place there. But this apprehension was rendered doubtful by the skin having undergone no change of colour; and it continued to show the same appearance several hours after the death of the patient, when I performed the operation, in order to satisfy myself as to the nature of the disease. The hernial tumour was then of the size of a turkey's egg, of an oblong figure, descending into the scrotum, and from four to five inches in length. I found the peritonæum thickened to one-fourth or one-third of an inch, adhering to the skin and to the omentum, which formed a large proportion of the hernia. The omentum was so condensed and indurated that it was difficult at first to distinguish it; and the testicle, exceedingly diminished in size, was enveloped in it. I observed no appearance of gangrene in the omentum, except at the lower part of it, which was black for about the extent of an inch, and reduced to a sort of ill-conditioned matter, rendered thin by the serous fluid, which, as I observed before, began to be formed about the twelfth day of the disease. So far I had been unable to discover any intestine, although it was plain that some part of that canal held an important share in the disease. I determined to divide the omentum to within within two inches of the abdominal ring, and I there found that it enveloped the intestine in its superior part as it did the testicle in its inferior part. This portion of intestine was strangulated by the ring for about an inch, but the appearance of it had undergone little alteration. This made me regret that the patient had not consented to undergo the operation even in some of the last days of the disease; as there is reason to believe that, even then, his life might have been saved by it.

Although this case does not afford the satisfaction of having been successful, it seems to prove that the treatment employed sufficed to support the life of the patient during a principal part of the time of the disease, and that it would finally have succeeded if his obstinacy had not prevented it.

I can also state, in favour of this treatment, another case more fortunate, in which I was consulted by my partner, Dr. Dunan. This practitioner was called to visit Mr. V. who had been attacked with the same disease. After having fruitlessly used attempts to reduce the hernia; and having tried the application of ice, he invited my assistance in the case. My opinion was, that it would be advisable to use the remedies employed in the former case in the fullest extent. Accordingly the patient was twice bled, he received injections, took three grains of opium, and was kept immersed in the warm bath for forty-two hours, at the end of which time the hernia was reduced of itself, without any external aid.

I know of no practitioner, before myself, who has undertaken to prolong the continuance in the warm bath so far; and M. Desault, although strongly attached to the remedy, only ordered it for a few hours, with the direction to repeat it afterwards. But I have supposed that no other limits ought to be placed to the use of the remedy but the weakness of the patient, who, if well nursed, and vigilantly watched, may be withdrawn from the bath on the least appearance of syncope.

MEMOIR

